# Changes in Gene Expression Associated with Collagen Regeneration and Remodeling of Extracellular Matrix after Percutaneous Electrolysis on Collagenase-Induced Achilles Tendinopathy in an Experimental Animal Model: A Pilot Study

**DOI:** 10.3390/jcm9103316

**Published:** 2020-10-15

**Authors:** José Luis Sánchez-Sánchez, Laura Calderón-Díez, Javier Herrero-Turrión, Roberto Méndez-Sánchez, José L. Arias-Buría, César Fernández-de-las-Peñas

**Affiliations:** 1 Department of Physical Therapy, Universidad de Salamanca, 37007 Salamanca, Spain; jlsanchez@usal.es (J.L.S.-S.); lauca@usal.es (L.C.-D.); ro_mendez@usal.es (R.M.-S.); 2 Physical Therapy Department, Mutua Accidentes Laborales, FREMAP, 37007 Salamanca, Spain; 3 Instituto de Neurociencias de Castilla y León, Universidad de Salamanca, 37007 Salamanca, Spain; mjaviht@usal.es; 4 Instituto Investigación Biomédica de Salamanca (IBSAL), Universidad de Salamanca, 37007 Salamanca, Spain; 5 Department of Physical Therapy, Occupational Therapy, Physical Medicine and Rehabilitation, Universidad Rey Juan Carlos (URJC), Alcorcón, 28922 Madrid, Spain; joseluis.arias@urjc.es; 6 Cátedra Institucional en Docencia, Clínica e Investigación en Fisioterapia: Terapia Manual, Punción Seca y Ejercicio Terapéutico, Universidad Rey Juan Carlos, Alcorcón, 28922 Madrid, Spain

**Keywords:** Achilles, percutaneous electrolysis, tendinopathy, gene expression

## Abstract

Percutaneous electrolysis is an emerging intervention proposed for the management of tendinopathies. Tendon pathology is characterized by a significant cell response to injury and gene expression. No study investigating changes in expression of those genes associated with collagen regeneration and remodeling of extracellular matrix has been conducted. The aim of this pilot study was to investigate gene expression changes after the application of percutaneous electrolysis on experimentally induced Achilles tendinopathy with collagenase injection in an animal model. Fifteen Sprague Dawley male rats were randomly divided into three different groups (no treatment vs. percutaneous electrolysis vs. needling). Achilles tendinopathy was experimentally induced with a single bolus of collagenase injection. Interventions consisted of 3 sessions (one per week) of percutaneous electrolysis or just needling. The rats were euthanized, and molecular expression of genes involved in tendon repair and remodeling, e.g., *Cox2*, *Mmp2*, *Mmp9*, *Col1a1, Col3a1, Vegf and Scx*, was examined at 28 days after injury. Histological tissue changes were determined with hematoxylin–eosin and safranin O analyses. The images of hematoxylin–eosin and Safranin O tissue images revealed that collagenase injection induced histological changes compatible with a tendinopathy. No further histological changes were observed after the application of percutaneous electrolysis or needling. A significant increase in molecular expression of *Cox2, Mmp9 and Vegf* genes was observed in Achilles tendons treated with percutaneous electrolysis to a greater extent than after just needling. The expression of *Mmp2, Col1a1, Col3a1, or Scx* genes also increased, but did not reach statistical significance. This animal study demonstrated that percutaneous electrolysis applied on an experimentally induced Achilles tendinopathy model could increase the expression of some genes associated with collagen regeneration and remodeling of extracellular matrix. The observed gene overexpression was higher with percutaneous electrolysis than with just needling.

## 1. Introduction

The prevalence of lower extremity injuries is extremely common in sport players but also in the general population [1]. Among injuries within the lower extremity, Achilles tendinopathy is highly prevalent and affects approximately 9% of recreational runners, 5% of professional athletes, and 5.6% of the sedentary population [2]. Tendinopathy can occur in tendons that receive excessive loads and can result in considerable pain and related-disability.

Cook et al. proposed a continuum model for human tendinopathy with three progressive and continuous potential stages: (1) initial reactive stage characterized by a noninflammatory proliferative tissue reaction with minimal collagen damage; (2) a second disrepair stage characterized by a greater tissue collagen disruption/tearing due to a failed attempt of the tendon to properly heal; in this stage proliferation of abnormal tenocytes and neovascularization can be present; (3) a degenerative stage characterized by further disruption of collagen, cell response, and the presence of neovascularization [3]. These authors emphasized the relevance of associating structural tendon changes with the clinical presentation of the patient due to the complexity in this association (or lack of). In fact, they proposed the tendinopathy as a hybrid reactive and degenerative pathology [3].

Based on current evidence, the pathophysiology of tendinopathy is not yet completely understood, different and multifactorial theories are plausible [4]. The first mechanism includes the presence of collagen degradation, disorganization and fragmentation of the cellular matrix, and increase of proteoglycan content [5]. The presence of neovascularization with the appearance and organization of a new non-functional vascular/neural network has also been proposed as a second theory [6]. These hypotheses have been confirmed by several experimental animal studies showing histopathological changes including proteoglycan accumulation, collagen fiber disorganization, increased blood vessel infiltration, increased cellularity, and cellular rounding in injured tendons [7,8]. In addition to histological changes, it has been observed that increases in gene expressions of proteoglycans, and disturbances in expression of matrix metalloproteinases (MMPs) and tissue inhibitors of MMPs (TIMPs) exist in pathological and healing tendons [9,10]. Current evidence supports that, regardless of the initiating event or the phase of the process, tendon pathology is characterized by a significant cell response to injury [3].

A better understanding of molecular changes accounting for tendinopathies could improve their management. In fact, the continuum model suggests that management may be optimized by tailoring therapeutic interventions to the stage of pathology and targeting the primary driver (cell activation) and inter-related alterations in tendon matrix integrity [3]. Currently, a wide range of interventions, i.e., extracorporeal shockwave therapy [11], injections [12], platelet-rich plasma [13], or exercises [14] have been proposed for the treatment of tendinopathies. These interventions can influence tendon biology by promoting its regeneration, but they are not able to influence the inflammatory process. An emerging strategy, i.e., percutaneous electrolysis, has been proposed for the management of tendinopathies [15]. Percutaneous electrolysis consists of the application of a continuous (galvanic) electrical current through a filament needle in a targeted tissue, usually tendon or muscle. There is preliminary evidence suggesting that application of percutaneous electrolysis is effective for patellar [16], plantar [17], elbow [18], or supraspinatus [19,20] tendinopathies; although more clinical trials are needed.

It is proposed that percutaneous electrolysis combines two physiological effects, a mechanical effect resulting from the needle insertion and a biological effect associated with the galvanic electrical current [15]. By inducing a non-thermal electrolytic reaction in the tendon, percutaneous electrolysis could potentially lead to a controlled inflammatory response, which may facilitate an organic reaction ultimately leading to the regeneration of the injured and healed tendon [15]. These hypotheses have been confirmed by recent studies. There is evidence suggesting that percutaneous electrolysis is able to induce an acute inflammatory reaction, mainly characterized by the presence of polymorphonuclear neutrophils and macrophages, in both healthy tendon [21] and collagenase-induced tendinopathy [22] in animals. The inflammatory response was most remarkable 7 and 14 days after the intervention [21,22]. Similarly, percutaneous electrolysis has been also able to induce an immediate and transitory vasodilation effect when applied to healthy animal tendons, which could facilitate the arrival of pro-inflammatory cells (essential for the regeneration of a healed tendon) and the drainage of nociceptive substances [23]. Although it seems that percutaneous electrolysis is able to promote an inflammatory response, the underlying mechanisms of this process are not yet known. In fact, a recent study found that changes in pH after the application of percutaneous electrolysis were relatively small and could not fully explain the neurophysiological and clinical effects of this intervention [24].

No previous study has investigated changes in expression of genes associated with collagen regeneration and remodeling of extracellular matrix in an experimentally injured tendon model after the application of percutaneous electrolysis. The use of animal models assists in understanding tendinopathies because they allow for the study of each progressive stage of the process in a controlled and reproducible environment. In the current animal pilot study, we used an experimentally induced tendinopathy model with a single collagenase injection in the Achilles tendon of rats to investigate gene expression changes after the application of percutaneous electrolysis. We hypothesized that the application of percutaneous electrolysis will be able to promote the healing process of an injured tendon by increasing the gene expression when compared with no intervention.

## 2. Methods

### 2.1. Experimental Design

This animal study was conducted at the Institute of Neurosciences of Castilla y Leon (University of Salamanca), and fulfilled the ethical requirements of and was approved by the Bioethics Committee of this institution (201899900014183). Procedures were conducted according to the European (Directive 2010/63/EU) and Spanish legislation (Royal Decree 53/2013; BOE 34/11370-421,2013) on experimental animal studies. All environmental conditions such as light or temperature during the experiment followed the regulations contained in Royal Decree 1201/2005. The study was designed to minimize the number of sacrificed rats.

Fifteen (n = 15) Sprague-Dawley male rats, aged 8 weeks, weighing approximately 250 g, were used. Rats were randomly divided into different groups according to the nature of the tissue (healthy vs. injured based on whether a tendinopathy was or not induced) or treatment applied (no treatment vs. percutaneous electrolysis vs. needling). The first groups (C, T1) were used to determine if changes induced by the collagenase injection were compatible with those observed in tendinopathies. The remaining groups were used for comparing the application of percutaneous electrolysis (T2 + PE) or control needling (T2 + N) versus no treatment (T2). Table 1 shows the distribution of the rats on the different groups.

### 2.2. Collagenase Experimentally Induced Tendinopathy

Rats were individually placed in a chamber. Anesthesia was induced with isoflurane (concentration 4–5%, oxigen 0.5–1 L/min). Subsequently, a collagenase injection was carried out with a 30-gauge needle 2 mm away from each rat Achilles osteotendinous junction. To induce Achilles tendon injury, a total of 250 units (30 μL) of collagenase (in 0.9% saline solution; bacterial type I; Sigma-Aldrich) previously filtered throughout a 0.22 μm Nalgene filter was injected [25]. We used one bolus injection of collagenase to mimic the acute reactive phase of a tendinopathy [25]. Rats serving as controls were injected with 30 μL of sterile saline solution (0.9% NaCl) to validate the injured model.

### 2.3. Percutaneous Electrolysis

**Figure 1:**
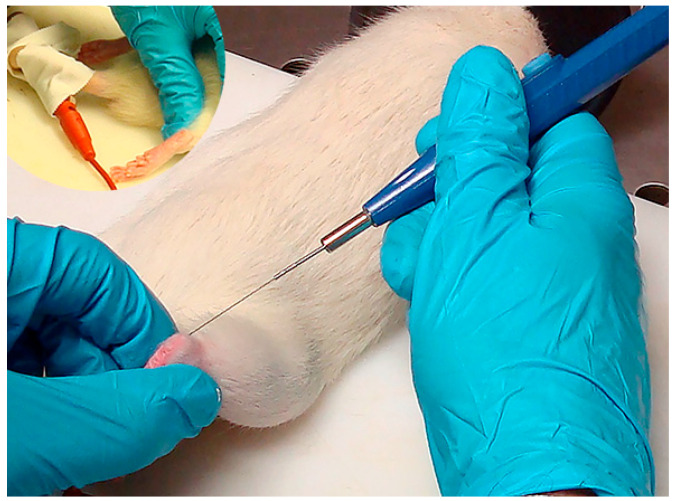
Application of Percutaneous Electrolysis (EPI© device) on the Achilles tendon of a rat. The positive electrode was placed on the tail of the rat with a cinch (figure on the left top corner).

The application of percutaneous electrolysis treatment was performed by using an approved EPI^®^ device (Epiadvanced, Barcelona, Spain), certified according to the Directive 93/42/CEE. The positive electrode was placed on the tail of the rat with a cinch. The negative electrode consisted of a sterile solid needle (0.3 mm diameter; 0.25 mm length; Agupunt, Barcelona, Spain). The needle was inserted at an angle of approximately 70° with the tip oriented towards the calcaneous bone of the rat (Figure 1).

**Figure 2:**
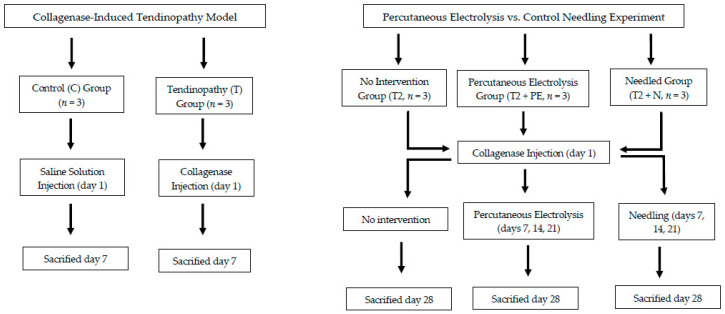
Flow Diagram summarizing the Experiment Groups.

Each percutaneous electrolysis session consisted of three punctures targeting the Achilles intra-tendon 2 mm away from the osteotendinous junction. The intensity of the continuous (galvanic) electrical current was set at 3 mA and applied for 4 s on each puncture [21,22]. A total of three sessions (a total of 9 punctures), once per week, were applied 7, 14, and 21 after the application of collagenase (Figure 2).

### 2.4. Control Needling

To determine if the observed changes were related to the electrical current applied during the percutaneous electrolysis and not just to the needle, a group of rats receiving the same needle insertion procedure was used as a control. Rats received needling for three sessions (3 punctures on 3 sessions) following the same procedure than for percutaneous electrolysis, i.e., 7, 14, and 21 after the application of collagenase, but without application of electrical current (Figure 2).

### 2.5. Tissue Sampling Preparation

Rats were euthanized by exsanguination while deeply anesthetized one week after collagenase injection in both groups to validate the collagenase model (C, T1) and 7 days after the last experimental session (28 days after collagenase injection) within the remaining groups (Table 1), and tissue samples were obtained.

The Achilles tendon was surgically removed from each rat leg using a sterile surgical knife. The skin was longitudinally cut from the calcaneous osteotendinous junction to the musculotendinous junction of the soleus and twin muscle.

Tissue samples for the histological analyses were embedded into an Optical Cutting Temperature compound (OTC) and frozen in methylbutane precooled to its freezing point using liquid nitrogen and stored at −80 °C until analysis.

Samples allocated to genetic analysis were embedded in 1 mL of Trizol^®^, frozen under liquid nitrogen, and stored at −80 °C until analysis.

### 2.6. Histological Analysis

Tissue samples were longitudinally cut into 10 μm sections on a cryostat (HM550, Thermo Fisher Scientific, Waltham, MA, USA) and mounted on poly-L-lysine-coated (Menzel-Glazer) microscopic slides. Samples were stained with hematoxylin-eosin and safranin O. The hematoxylin-eosin staining allows observation of collagen structures and identification of tenocyte nuclei and muscular fibres, whereas safranin staining allows the identification of cartilaginous tissue cells.

Stained tissue samples were observed using an Olympus AX-70 microscope coupled with an Olympus Apogee digital camera. Images brightness and contrast were adjusted using Adobe Photoshop CS4.

### 2.7. Genetic Analysis

Ribonucleic acid (RNA) was extracted using a modified version of the procedure described by Chomczynsky and Sacchi [26]. Total RNA was sequentially extracted using 1 mL of Trizol^®^ per 100 mg of tissue and purified using a commercial kit (RNeasy Mini Kit^®^ Qiagen, Hilden, Germany). The RNA was quantified and its quality assessed by using an Agilent 2100 Bioanalyzer. Only samples with an RNA integrity number (RIN) >8.0 were used.

Complementary DNA (cDNA) was synthesized using messenger RNA (mRNA) contained in purified RNAs via a retrotranscription enzymatic reaction using the kit ImProm-IITM Reverse Transcripción System (Promega). Total RNA (2 µg), primed with a mix of oligo-dT and random hexamer primers, was reverse-transcribed into cDNA at 37 °C for 2 h using the first-strand cDNA synthesis kit (Promega Corporation, Madison, WI, USA) into a 20 µL volume, and stored at −20 °C of temperature until use, according to the manufacturer’s instructions. In all cases, a reverse transcriptase negative control was performed to rule out genomic DNA contamination.

The DNA was amplified using polymerase chain reaction (PCR) and quantified using real time quantitative PCR (RT-qPCR). Before quantification of amplified DNA samples, efficiency analysis for each primer (specific for each targeted gene) was performed (Table 2). These analyses allowed us to establish the expression dynamic range for each primer. The RT-qPCR was performed following the SYBR-Green method [27] and using the primers listed in Table 2. Three genes (β-actin, β-act; 60 S ribosomal protein L19, Rpl19; and glyceraldehyde 3-phosphate dehydrogenase, Gapdh) were selected as internal standards to allow RT-qPCR data normalization. The NormFinder software was used to calculate the intra- and inter-group variations in their expressions. Finally, the mean of the threshold cycle (Ct) value and the primer efficiency value of Gapdh were used for normalization, as Gapdh was the most stable gene.

The comparative Ct method was used for presenting quantitative data [28]. Following removal of outliers, raw fluorescence data were used to determine PCR amplification efficiency (*E*) as follows:

$$E=\left[10^{\left(-\frac{1}{slope}\right)}100\right]$$

All amplifications had an *E* value of 100 ± 10%, an *E* value close to 100% being an indicator of efficient amplification.

$$FC=E^{-\Delta \Delta Ct}$$

The statistical significance level of qPCR analysis was determined using a one-sample *t*-test for each gene, testing whether |FC| > 1 is significant (*p* < 0.05).

The targeted genes for the current study included the cyclooxygenase 2 (*Cox2*) gene, which triggers inflammatory processes and the expression of some proteinases such as matrix metalloproteinases (MMPs) and tissue inhibitors of metalloproteinases (TIMPs). The expression of metalloproteinase 2 and 9 (*Mmp2 and Mmp9*), Collagen type I alpha 1 chain (*Col1a1*) and collagen type III alpha 1 chain (*Col3a1*) genes were also evaluated, all components of the tendon extracellular matrix. We also analyzed growth factor genes, particularly the vascular endothelial growth factor (*Vegf*) gene, which is able to promote angiogenesis and vascular permeability, as well as the Scleraxis gene (*Scx*), a relevant gene key in the development of fetal tenocytes and in tendon remodeling processes in adults.

## 3. Results

### 3.1. Changes after Collagenase Injection (C vs. T1)

**Figure 3:**
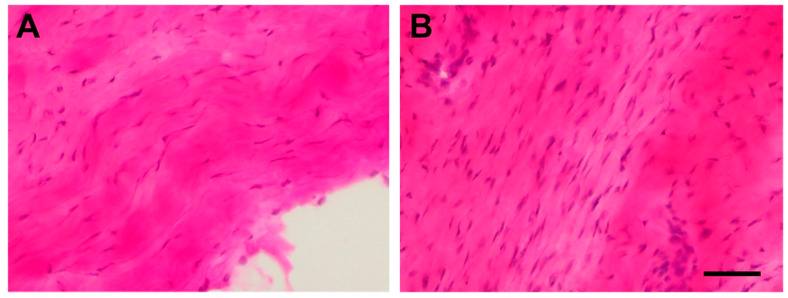
Stained longitudinal images with hematoxylin–eosin of a normal Achilles tendon (**A**) and of an injured Achilles tendon of rats euthanized after one week after injection of collagenase (**B**). The scale bar is at 50 μm.

**Figure 4:**
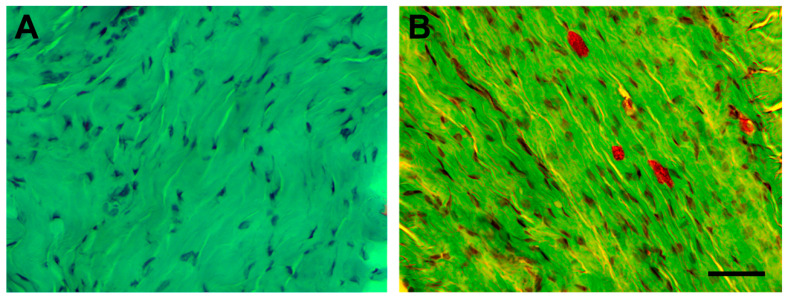
Stained longitudinal images with Safranin O of a normal Achilles tendon (**A**) and of an injured Achilles tendon of rats euthanized after one week after injection of collagenase showing the presence of fibrocartilaginous tissue (**B**). The scale bar is at 50 μm.

The images of hematoxylin–eosin and Safranin O stained tissue corresponding to the injured tendon of rats euthanized one week after the injection of collagenase (induced tendinopathy, T1) showed substantial damage as compared with tissue images from the tendon acting as control (C) one week after the injection of sterile saline solution (Figure 3 and Figure 4). In particular, increased tenocyte number, misalignment of collagen fibres, and loss of extracellular matrix organization as well as increased vascularization (Figure 3B) was seen in the experimentally induced Achilles tendinopathy. Safranin O staining images also identified the presence of fibrocartilaginous tissue (Figure 4B).

**Figure 5:**
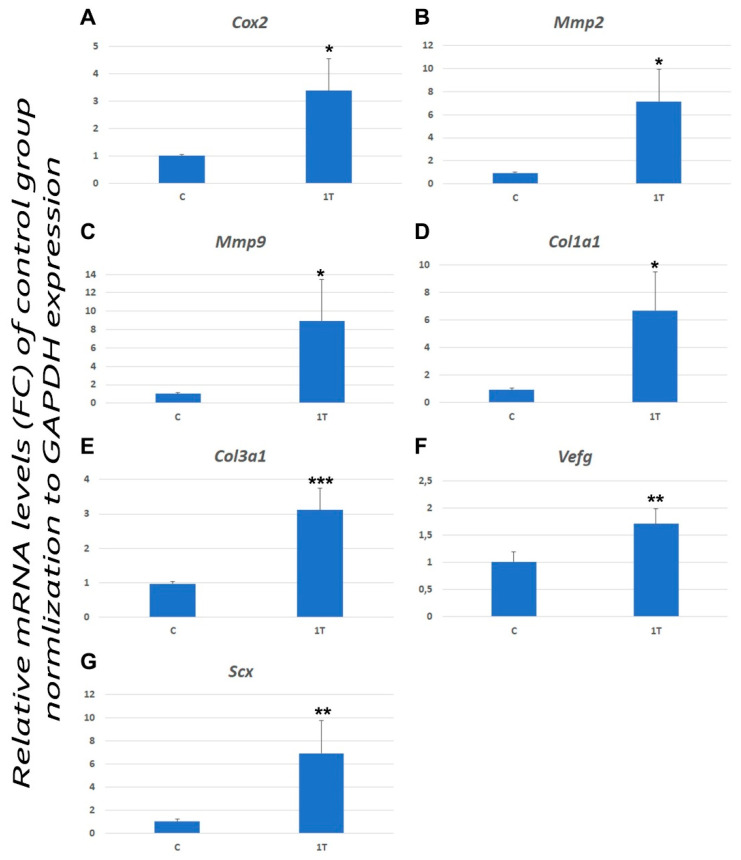
Levels of expression of *Cox2* (**A**), *Mmp2* (**B**), *Mmp9* (**C**), *Col1a1* (**D**), *Col3a1* (**E**), *Vegf* (**F**) and *Scx* (**G**) genes in an healthy control Achilles tendon (C) and experimentally induced tendinopathy tendon (T1). Data were quantified and normalized by using the RT-qPCR procedure. Each bar represents the fold change (FC) ± standard deviation (SD) of the normalized values. *** *p* < 0.001; ** *p* < 0.01; * *p* < 0.05.

In addition, a significant increase in the expression of *Cox2, Mmp2, Mmp9, Col1a1, Col3a1, Vegf* and *Scx* genes (Figure 5) was also observed within the experimentally induced tendinopathy group compared with the control group (Table 3).

### 3.2. Percutaneous Electrolysis on Experimentally Induced Achilles Tendon

**Figure 6:**
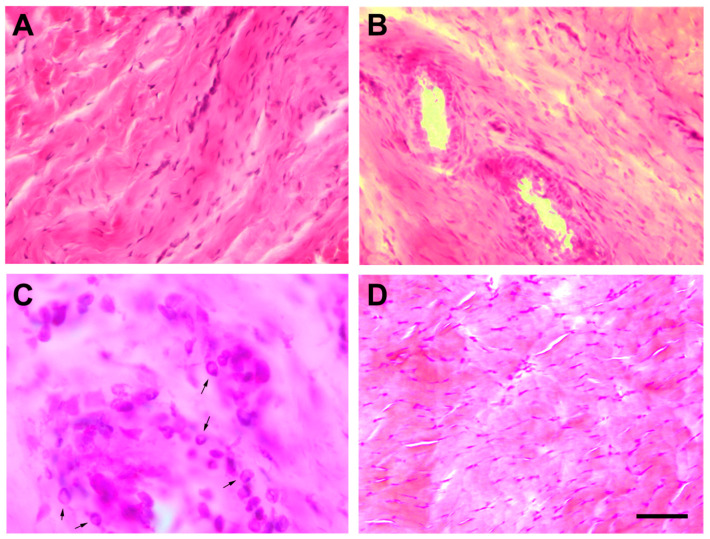
Stained longitudinal images with hematoxylin–eosin of injured Achilles tendon with collagenase injection by group: no intervention (**A**), treated with percutaneous electrolysis (**B**,**C**), or treated needling (**D**). Arrows in C shows several leucocyte cells. The scale bar is at 50 μm (**A**,**B**,**D**) and 10 μm (**C**).

The images of hematoxylin–eosin- and Safranin O-stained tissue revealed similar histological changes, e.g., increase of tenocytes, loss in extracellular matrix organization, misalignment of collagen fibers and striking neovascularization, between injured Achilles tendons subjected to no treatment (T2, Figure 6A), Achilles tendon treated with percutaneous electrolysis (T2 + PE, Figure 6B,C) and injured tendon treated with needling (T2 + N, Figure 6D). Nevertheless, tendinous tissues of injured Achilles tendons treated with percutaneous electrolysis showed slightly more signs of inflammation (presence of leukocytes) than others, but this was not significant.

**Figure 7:**
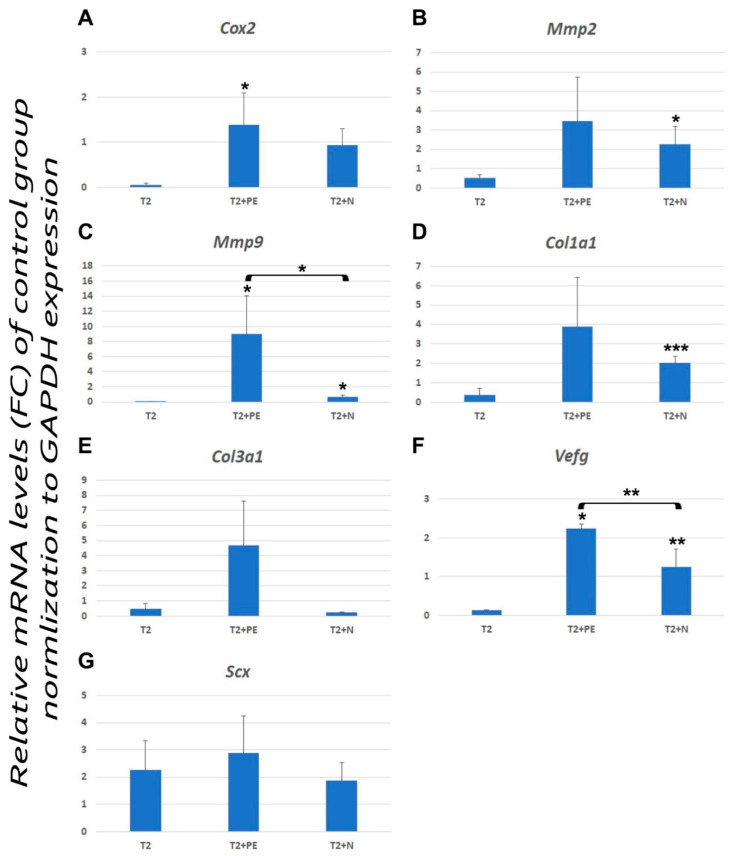
Levels of expression of *Cox2* (**A**), *Mmp2* (**B**), *Mmp9* (**C**), *Col1a1* (**D**), *Col3a1* (**E**), *Vegf* (**F**) and *Scx* (**G**) genes in an injured Achilles tendon with collagenase injection by group: no intervention (T2), percutaneous electrolysis (T2 + PE) and needling (T2 + N). Data were quantified and normalized by using the RT-qPCR procedure. Each bar represents the fold change (FC) ± standard deviation (SD). *** *p* < 0.001; ** *p* < 0.01; * *p* < 0.05.

A significant increase in the expression of *Cox2, Mmp9 and Vegf* genes was observed in the injured Achilles tendons treated with percutaneous electrolysis (Figure 7A,C,F; Table 4) in comparison with Achilles tendons without treatment (T2). Expression of *Mmp2, Col1a1, Col3a1 and Scx* genes was also higher in the group treated with percutaneous electrolysis, but this did not reach statistical significance (Figure 7B,D,E,G; Table 4). The application of a needling procedure only on injured Achilles tendons (T2 + N) also increased the expression of *Mmp2, Mmp9, Col1a1 and Vegf* genes when compared to non-treated injured Achilles tendons (T2), but to a lesser extent than after percutaneous electrolysis (Table 4).

## 4. Discussion

This animal study found that application of percutaneous electrolysis in experimentally induced Achilles tendinopathy with collagenase injection increased the expression of some genes related to collagen regeneration and tissue remodeling of extracellular matrix, without significant induction of further histological changes. Gene overexpression was higher than that observed after application of a needling procedure. In particular, we observed changes in three (i.e., *Cox2, Mmp9, and Vegf*) out of seven of the gene expressions analyzed.

### 4.1. Experimentally Induced Tendinopathy with Collagenase Injection

Before determining the effect of percutaneous electrolysis, we determined if the experimentally induced model with collagenase injection was able to mimic acute tendinopathy changes. We found an increase in the number of tenocytes, misalignment of collagen fibers, loss of extracellular matrix organization, and increased vascularization in experimentally injured Achilles tendons of rats. The presence of these changes agrees with previous experimental studies in animal models [29,30,31].

An increase in the expression of *Cox2, Mmp2, Mmp9, Col1a1, Col3a1, Vegf and Scx* genes is also consistent with data reported in previous human studies [10,32,33]. Riley found an overexpression of the collagen genes *Col1a1 and Col3a1* in human tendinous tissue was correlated with extracellular matrix organization loss in the acute phase [34]. Past studies have also reported an overexpression of these pro-inflammatory genes as well as significant increases within the expression of extracellular matrix metalloproteinases (MMPs) such as *Mmp2 and Mmp9* [35,36]. Finally, an overexpression of *Vegf* has been also reported to be present in tendinopathies and is attributed to an increase in vascularization due to the creation of new, but not functional, blood vessels [37,38].

Previous studies have also reported overexpression of proinflammatory genes such as *Cox2* during the healing process after a tendon injury [39,40]. Another important gene related to tendon remodeling is *Scx*, which also plays a relevant role in triggering *Col1a1* gene transcription [41,42]. This generalized gene overexpression has been related to the synthesis of new collagen, necessary to restore the tendon extracellular matrix organization. We found overexpression of all these genes in experimentally induced Achilles tendinopathy 7 days after injection of collagenase in the rats.

In conclusion, tissue images of Achilles tendons of rats euthanized one week after a collagenase injection showed histological and genetic changes that were characteristic of human tendinopathy, which was also observed in previous experimentally induced animal models. These results suggest that the collagenase-induced model used in our study was adequate.

### 4.2. Gene Expression Changes after Percutaneous Electrolysis

It has been suggested that percutaneous electrolysis triggers an acute inflammatory response in the tendon leading to activation of restoring and healing tissue mechanisms [15]. The hypothesis of an inflammatory response after the application of percutaneous electrolysis has been confirmed in animal models [21,22]. This study is the first experimental animal model investigating changes in gene expression in an injured tendon. We observed a statistically significant increase in expression of *Cox2, Mmp9 and Vegf* genes, but a non-significant increase in *Mmp2, Col1a1, Col3a1 and Scx* genes after the application of three sessions of percutaneous electrolysis in comparison with no treatment. The application of a needling procedure also resulted in increases in gene expression, but to a lesser extent than with percutaneous electrolysis.

Previous studies have reported an overexpression of *Cox2* [43], *Mmp2* [44], *Mmp9* [45], *Col1a1* [39], *Col3a1* [46], *Vegf* [47,48], and *Scx* [49] genes at the initial stage of an inflammatory process after acute tendon injury but with a progressive decrease during the following 7–28 days. This time trend suggests that these proinflammatory genes and metalloproteinases participate in the initial (acute) degradation of extracellular matrix, but their long-term overexpression plays a relevant role within collagen regeneration and extracellular matrix remodeling during tendon healing [39,43,44,45,46,47,48,49]. In this pilot study, rats with experimentally induced Achilles tendinopathy treated with percutaneous electrolysis exhibited a generalized increase in gene expression in comparison with the needling intervention or no treatment at 28 days after collagenase injection; however, only overexpression of *Cox2, Mmp9 and Vegf* genes was statistically significant. Although *Mmp9, Col1a1, Col3a1 and Scx* gene expression also increased after percutaneous electrolysis application, the changes did not reach statistical significance. A possible explanation is that rats were euthanized 28 days after collagenase injection and 7 days after the last treatment session, a potential time window for the natural decrease in gene expression [43,44,45,46,47,48,49]. A second potential reason explaining the lack of statistical significance in the *Mmp2, Col1a1, Col3a1 and Scx* genes could be related to the small number of rats included in this pilot study, which could lead to underpowered results.

The fact that greater gene expression changes were observed after the application of percutaneous electrolysis than with just the needling application provides preliminary evidence suggesting that the application of continuous (galvanic) current leads to a greater (or faster) increase of gene expression than with application of a needling procedure. A facilitated-gene overexpression could promote collagen regeneration and extracellular matrix remodeling in healed tendons supporting the potential clinical application of this intervention for the treatment of tendinopathies.

An important finding of the current study was that histological images did not reveal significant changes between experimental groups, suggesting that the application of percutaneous electrolysis or needling promoted overexpression of genes associated with tendon healing but without inducing further histological damage to the tendon. Nevertheless, these findings should be considered in the scenario of the current study, since only three sessions of percutaneous electrolysis were applied, which may have been not enough to observe changes within the extracellular tendon matrix, as well as the small number of rats included. It is possible that a greater number of sessions or a greater electrical current voltage could lead to structural tendon changes and higher gene overexpression.

### 4.3. Clinical Implications

Our results have potential implications for clinical practice. All genes investigated in this study were present at the initial stage of the inflammatory process after a healed tendon but they progressively decrease over the following 7–28 days [43,44,45,46,47,48,49]. A long-term overexpression, as observed after the application of percutaneous electrolysis, may play a role for promoting collagen regeneration and extracellular matrix remodeling in a healed tendon. Nevertheless, the current study did not assess changes in protein levels; therefore, we cannot conclude that gene overexpression observed after the application of percutaneous electrolysis was able to produce better regeneration of the tendon matrix and, hence, to improve collagenase-induced tendinopathy. Future studies should investigate changes in protein levels after the application of percutaneous electrolysis. Current and previous data suggest that application of percutaneous electrolysis can trigger an initial biological (inflammatory) response in the tendon, preparing the tissue for better loading. In fact, controlled tendon loads with exercise will be clearly needed to be combined with percutaneous electrolysis to facilitate the process of collagen tissue proliferation, thus improving the biomechanical properties of the tendon. This hypothesis is partially supported by randomized clinical trials showing that combining percutaneous electrolysis with a progressive exercise program represents a promising management strategy for tendinopathies of the elbow [19], shoulder [21], or knee [17]. Future trials determining the biological effects of combing percutaneous electrolysis with active therapies such as exercise would help to elucidate the underlying mechanisms of the clinical benefits of this intervention.

### 4.4. Limitations

Finally, we should recognize potential limitations to the current study. First, the number of rats on each group was small, which could lead to a lack of power in some comparisons. Therefore, this study should be considered as a pilot study. Due to the lack of previous studies investigating changes in gene expression, it was not possible to make “a priori” calculation of the most appropriate sample size. Considering the data obtained in this pilot study, an estimated sample size calculation was conducted to get statistical significance in all the analyzed genes. Assuming a pooled standard deviation ranging from 0.1 to 2.1 units, a two-tailed alpha level (α) of 0.05 and a desired power (1-β) of 80%, it was estimated that a sample of 5 to 7 rats per group would be required to detect between-group differences from 1.0 to 3.0 units. Nevertheless, it is important to consider that we found significant differences in three out of seven gene expressions with the current sample of rats. Second, it seems that the dosage of the galvanic electric current plays a relevant role in the inflammatory response elicited by percutaneous electrolysis. Future studies should investigate changes in gene expression with different dosages. In fact, clinicians apply this intervention with different doses and frequencies of sessions depending on the clinical presentation of the patient. Therefore, current results should be considered in the investigated scenario. Finally, the application of percutaneous electrolysis is usually ultrasound-guided [16,17,18,19,20,21]. In the current study, the technique was not ultrasound-guided, which could have influenced the results.

## 5. Conclusions

This experimental animal pilot study found that the application of three sessions (once a week) of percutaneous electrolysis into an experimentally induced Achilles tendinopathy with collagenase injection increases the expression of some genes related to collagen regeneration and remodeling of extracellular matrix, particularly the *Cox2, Mmp9 and Vegf* genes. These changes were greater than those observed after the application of a needling procedure. No additional histological changes were found with either intervention, suggesting that both interventions did not provide further damage into the tendon. Current results should be considered as preliminary and need to be confirmed in future studies.

## Figures and Tables

**Table 1:** Random Distribution of Rats with Experiment Procedures by Group.

Group Number	Group	Number of Rats	Collagenase Injection	Day Collagenase Injection	PE Treatment	Days of PE Intervention	Needling Treatment	Day of Needling Treatment	Day of Sacrifice
**Validation of Collagenase-Induced Tendinopathy**									
1	C *	3	No		No		No		7
2	T1 ^#^	3	Yes	1	No		No		7
**Experimental Study**									
3	T2 ^#^ (no treatment)	3	Yes	1	No		No		28
4	T2 + PE	3	Yes	1	Yes	7, 14, 21	No		28
5	T2 + N	3	Yes	1	No		Yes	7, 14, 21	28

C: control; T: Tendinopathy model; PE: Percutaneous Electrolysis; N: Needling. * Control group (C) was injected with saline solution; ^#^ Experimentally induced tendinopathy groups (T1 and T2) were injected with collagenase. T1: Experimentally induced tendinopathy group used for confirming the collagenase model. Rats were sacrificed 7 days after the collagenase injection. T2: Experimentally induced tendinopathy groups randomly assigned to no treatment, percutaneous electrolysis (PE) or needling (N). Rats were sacrificed 28 days after the collagenase injection.

**Table 2:** Oligonucleotide Primer Sequences used for qPCR.

Gene Name	GenBank Number *	Oligonucleotide Primer Sequence (Forward; 5′-3′)	Forward Location *	Oligonucleotide Primer Sequence (Reverse; 3′-5′)	Reverse Location	Amplicon Size	Primer Efficiency
*Cox2*	S67722	CCCATGGGTGTGAAAGGAAA	566–586	GGGATCCGGGATGAACTCTC	636–655	90	1.96
*Col1a1*	NM_053304	GCCTCAGCCACCTCAAGAGA	3681–3701	GGCTGCGGATGTTCTCAATC	3801–3820	140	2.06
*Col3a1*	NM_032085	CCAGGACAAAGAGGGGAACC	1194–1213	CCATTTCACCTTTCCCACCA	1277–1297	103	1.99
*Mmp2*	NM_031054	ACACCTGACCTGGACCCTGA	615–634	TTCCCCATCATGGATTCGAG	700–719	105	1.98
*Mmp9*	NM_031055	GCAGGGCCCCTTTCTTATTG	1659–1679	CTGGCCTGTGTACACCCACA	1769–1788	130	2.02
*Vegf*	NM_031836	GCAATGATGAAGCCCTGGAG	1271–1290	GCTGGCTTTGGTGAGGTTTG	1338–1357	87	1.97
*Scx*	NM_001130508	GAGAACACCCAGCCCAAACA	700–720	CGAATCGCCGTCTTTCTGTC	769–788	89	2.00
*β-act*	NM_031144	AGCCATGTACGTAGCCATCC	415–434	ACCCTCATAGATGGGCACAG	510–529	115	1.98
*Gapdh*	NM_017008	ACATGCCGCCTGGAGAAACCT	805–824	GCCCAGGATGCCCTTTAGTGG	874–894	90	1.96
*Rpl19*	NM_031103	TCGCCAATGCCAACTCTCGTC	123–143	AGCCCGGGAATGGACAGTCAC	191–211	89	2.07

*: Oligonucleotide location within its corresponding GenBank sequence. *Scx*: Scleraxis; *Vegf*: vascular endothelial growth factor; *Cox2*: cyclooxygenase 2; *Col1a1*: collagen type I alpha 1 chain; *Col3a*1: Collagen type III alpha 1 chain; *Mmp2*: Matrix metalloproteinase 2; *Mmp9*: Matrix metalloproteinase 9: *β-act*: Beta actin; *Gapdh*: Glyceraldehyde-3-phosphate; *Rpl19*: 60 S ribosomal protein L19.

**Table 3:** Gene Expression Levels in healthy (C) and experimentally injured (T1) Achilles Tendons of Rats (sacrificed at 7 days after the injection).

Gene Name	Group	Fold Change	Standard Deviation	*p*-Value
*Cox2*	Healthy tendon (C)	1.001	0.049	
	Injured tendon (T1)	3.367	1.184	0.02
*Mmp2*	Healthy tendon (C)	0.941	0.106	
	Injured tendon (T1)	7.137	2.783	0.02
*Mmp9*	Healthy tendon (C)	1.004	0.128	
	Injured tendon (T1)	8.972	4.478	0.015
*Col1a1*	Healthy tendon (C)	0.923	0.158	
	Injured tendon (T1)	6.691	2.801	0.025
*Col3a1*	Healthy tendon (C)	0.967	0.062	
	Injured tendon (T1)	3.119	0.631	0.004
*Vefg*	Healthy tendon (C)	1.011	0.183	
	Injured tendon (T1)	1.708	0.274	< 0.001
*Scx*	Healthy tendon (C)	1.023	0.221	
	Injured tendon (T1)	6.935	2.851	0.004

*Cox2*: cyclooxygenase 2; *Mmp2*: Matrix metalloproteinase 2; *Mmp9*: Matrix metalloproteinase 9; *Col1a1*: collagen type I alpha 1 chain; *Col3a*1: Collagen type III alpha 1 chain; *Vegf*: vascular endothelial growth factor; *Scx*: Scleraxis.

**Table 4:** Gene Expression Levels in Experimentally induced Tendinopathy Achilles Tendon of Rats (T2) according to intervention (no intervention, percutaneous electrolysis and needling).

Gene Name	Group	Fold Change	Standard Deviation	*p*-Value (T2 vs. T2 + PE/T2 + N)	*p*-Value (T2 + PE vs. T2 + N)
*Cox2*	T2 (28 days)	0.056	0.031		
	T2 + PE	1.392	0.697	0.03 *	
	T2 + N	0.937	0.365	0.05	0.3
*Mmp2*	T2 (28 days)	0.523	0.163		
	T2 + PE	3.468	2.251	0.07	
	T2 + N	2.275	0.911	0.03 *	0.3
*Mmp9*	T2 (28 days)	0.040	0.006		
	T2 + PE	8.977	5.069	0.02 *	
	T2 + N	0.658	0.225	0.01 *	0.02
*Col1a1*	T2 (28 days)	0.364	0.345		
	T2 + PE	3.875	2.547	0.07	
	T2 + N	2.009	0.336	<0.001 ***	0.25
*Col3a1*	T2 (28 days)	0.452	0.348		
	T2 + PE	4.654	2.972	0.06	
	T2 + N	0.219	0.026	0.15	0.05
*Vefg*	T2 (28 days)	0.133	0.001		
	T2 + PE	2.229	0.117	0.025 *	
	T2 + N	1.248	0.462	0.005 **	0.007 **
*Scx*	T2 (28 days)	2.256	1.087		
	T2 + PE	2.886	1.373	0.5	
	T2 + N	1.860	0.672	0.45	0.25

*Cox2*: cyclooxygenase 2; *Mmp2*: Matrix metalloproteinase 2; *Mmp9*: Matrix metalloproteinase 9; *Col1a1*: collagen type I alpha 1 chain; *Col3a*1: Collagen type III alpha 1 chain; *Vegf*: vascular endothelial growth factor; *Scx*: Scleraxis; *** *p* < 0.001; ** *p* < 0.01; * *p* < 0.05.
